# Functionalization of Gold Nanoparticles with Ru-Porphyrin and Their Selectivity in the Oligomerization of Alkynes

**DOI:** 10.3390/ma15031207

**Published:** 2022-02-05

**Authors:** Francesca Limosani, Hynd Remita, Pietro Tagliatesta, Elvira Maria Bauer, Alessandro Leoni, Marilena Carbone

**Affiliations:** 1 Department of Information Engineering, Polytechnic University of Marche, Via Brecce Bianche, 1, 60131 Ancona, Italy; 2 INFN-National Laboratories of Frascati, Via Enrico Fermi, 40, Frascati, 00044 Rome, Italy; 3 Institut de Chimie Physique, UMR 8000 CNRS, Université Paris-Saclay, 91405 Orsay, France; hynd.remita@universite-paris-saclay.fr; 4 Department of Chemical Sciences and Technologies, University of Rome Tor Vergata, Via della Ricerca Scientifica, 1, 00133 Rome, Italy; alessandro.leoni@uniroma2.it (A.L.); carbone@uniroma2.it (M.C.); 5 Institute of Structure of Matter (CNR-ISM), Italian National Research Council, Via Salaria km 29.3, Monterotondo, 00015 Rome, Italy; elvira.bauer@ism.cnr.it

**Keywords:** gold nanoparticles, metalloporphyrin, oligomerization, arylalkynes, catalysis

## Abstract

Gold nanoparticles (AuNPs) were functionalized by ruthenium porphyrins through a sulfur/gold covalent bond using a three-steps reaction. The catalyst was characterized by scanning electron microscopy (SEM) and thermogravimetric analysis (TGA) in order to control the binding of ruthenium porphyrin on AuNPs’ surface. The catalyst was tested and compared with an analog system not bound to AuNPs in the oligomerization reaction using 1-phenylacetylene as the substrate.

## 1. Introduction

Gold nanoparticles (AuNPs) are attracting the attention of a large community of scientists because of their tunable electronic structures that generate useful characteristics such as size-related and shape-related optoelectronic properties [1,2,3]. large surface-to-volume ratio, excellent biocompatibility, and low toxicity [4,5,6], which stimulated their applications in several fields such as biotechnology [7,8], sensing [9], and catalysis [10].

Properties and applications of AuNPs can be tuned by functionalization, which allows the imprinting of a hydrophilic or a hydrophobic character, varies hindrance, and influences photoluminescence. Functionalization is usually carried out by capping AuNPs with labile ligands (citrates, thiols, or other adsorbed ligands), subsequently displaced by thiols through a location with ligand exchange reaction to synthesize monolayer-protected AuNPs, thus allowing the spontaneous binding of organic molecules or biomolecules through the formation of –S–Au bonds [11,12,13].

Hybrid architectures composed of both porphyrin/metalloporphyrin and different materials such as organic or inorganic nanoparticles [14,15] and carbon-based-nanomaterials [16], obtained by a bottom-up approach, are well suited for the formation of functional nanostructures [17] that display structural control and synergic functionalities. These preparation methods will result in the formation of unique materials with properties fundamental for the development of compounds that find wide space in several fields such as optics [18,19,20,21,22]; electronics [23]; photodynamic therapy [24]; materials able to enhance light absorption [25]; and catalysis [26].

Ruthenium porphyrins, properly functionalized, represent suitable candidates to be used as organic units to bind on AuNPs’ surface. Some different synthetic methods have been proposed to obtain these assemblies, depending on the porphyrin functional group used as a linker for AuNPs [27,28]. These networks represent the combination of different moieties with various functionalities in order to obtain a final composite material that has more complex functions to be employed in the field of catalysis.

Metalloporphyrins are well known for their catalytic properties [29] in many important organic reactions, such as, for example, the oxidation of organic substrates [17,30,31,32]; the cyclopropanation of olefins [33,34,35,36]; the carbonyl ylide/1,3-dipolar cycloaddition reactions of a-diazoketones [37]; the insertion of carbene into the S–H bond [38,39]; the amination of hydrocarbons [40,41]; and the olefination of aldehydes [42].

The oligomerization reaction [26,43,44,45] is an important test to obtain information on the conjugated compound because it can provide different, although limited products, alkyne scaffolds, which are considered the most useful building blocks for a wide variety of applications such as organic light emitters [46,47], discotic liquid crystals [48,49], high-porosity materials [50,51], or tools to design organic units in synthetic and material chemistry [52,53].

In the present study, we investigated the functionalization of AuNPs with a Ru-thiomethyl-tetraphenylporphyrin (Ru-TPP-CH_2_-SH) and compared the selectivity of coupled compound Ru-TPP-CH_2_S-AuNPs to separate moieties for the oligomerization of phenylacetylene.

The aim of this investigation is to determine whether the conjugated compound Ru-TPP-CH_2_S-AuNPs is stable and its catalytic properties prevail over metalloporphyrin without AuNPs.

The synthetic strategy required an ad hoc synthesis of Ru tetraphenylporphyrin, substituted with a thiomethyl group in para-position of one of the phenyl rings (Ru-TPP-CH_2_-SH) and subsequent functionalization with monodispersed gold nanoparticles. The strategy adopted for the synthesis of Ru-TPP-CH_2_-SH is a three-stepped one based on the substitution of the acetyloxy group of 5-(4′-acetyloxymethylphenyl)-10,15,20-triphenylporphyrin by the thioacetate group, metalation, and subsequent deprotection of the thioacetate to yield the corresponding thiol group. The 30 nm diameter, monodispersed citrate capped AuNPs were obtained by radiochemical methods and subsequently used for functionalization according to a recently standardized method [54]. This is a two-phases reaction, where the citrate substitution with thiolated porphyrin occurs at the interphase between the aqueous and the organic solution, using acetone as transfer agent. The obtained catalyst Ru-TPP-CH_2_S-AuNPs was, then, tested and compared with the not-bound Ru-TPP-CH_2_-SH in an oligomerization reaction using phenylacetylene as a substrate.

## 2. Materials and Methods

### 2.1. Materials and Equipment

Compounds 5-(4′-acetyloxymethylphenyl)-10,15,20-triphenylporphyrin (**1**), 5-(4′-acetylthiomethylphenyl)-10,15,20- triphenylporphyrin (**2**), ruthenium(II) 5-(4′-acetylthiomethylphenyl)- 10,15,20-triphenylporphyrin (**3**), and ruthenium(II) 5-(4′-sulfanylmethylphenyl)- 10,15,20-triphenylporphyrin (**4**) were synthesized and characterized as reported [55].

The formation of dimerization and trimerization products of phenylacetylenes was measured by gas chromatography (GC) analyses, with a Focus Thermo Fisher Instrument (Waltham, MA, USA), using helium as the carrier gas (35 cm/s). A Restek MXT-5 column (length 15 m; i.d. 0.28 mm; low-polarity stationary phase) was used. The gas chromatographic conditions were as follows: initial temperature, 70 °C for 2 min; temperature increase rate, 40 °C/min; final temperature, 320 °C; injector temperature, 320 °C; detector temperature, 350 °C.

The compounds are detected by flame ionization detector (FID).

The surface morphology and diameter of AuNPs was determined with an FE-SEM, Field Emission Scanning Electron Microscope SUPRA TM 35, Carl Zeiss SMT, Oberkochen (Germany), operating at 7 kV. The diameter of AuNPs was estimated using Image J and the histogram of particle size was elaborated with Origin Pro program and fitted using a Gaussian function to determine the averaged particle size. For the estimation of the particle size, we used 4 microscope images, analyzing the size of 55–65 particles per image.

TGA measurements were carried with a TA-Instruments Q500 under N_2_ flow at a heating rate of 10°/min.

### 2.2. Synthesis

#### 2.2.1. Synthesis of AuNPs

**Scheme 1:**
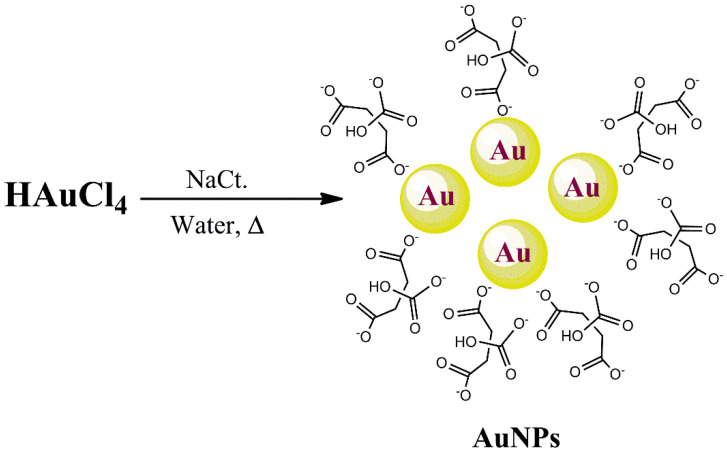
Synthetic pathway for obtaining AuNPs.

The synthesis of the AuNPs was performed by a reaction of HAuCl_4_ with trisodium citrate (NaCt), which also acts as a capping agent (Scheme 1). In a typical synthesis, 50 mL of 0.25 mM HAuCl_4_ was refluxed in water under constant and vigorous stirring.

Then, 1 mL of 34.0 mM (1.0 wt.%) trisodium citrate (NaCt) was added. The colour of the solution turns from light yellow to grey and finally to red. The red colour corresponds to the formation of AuNPs (with a plasmon around 520 nm). The reaction was carried out until the colour of the suspension did not undergo any further changes. The resulting suspension/solution was cooled to room temperature.

#### 2.2.2. Synthesis of Ru-TPP-CH_2_-SH (**4**)

The strategy adopted for the synthesis of Ru-TPP-CH_2_-SH porphyrin used for binding on the AuNPs surface in order to form the new Ru-TPP-CH_2_S-AuNPs catalyst is a three-step reaction.

The Ru-TPP-CH_2_S-AuNPs catalyst investigated in this work has never been considered for catalytic applications. All intermediate compounds have been synthesized and characterized according to the literature [55], as highlighted in Section 2.1.

The starting material 5-(4′-acetyloxymethylphenyl)-10,15,20-triphenylporphyrin was obtained according to the literature method [56].

The first step was the substitution of the acetyloxy group of the 5-(4′-acetyloxymethylphenyl)-10,15,20-triphenylporphyrin (**1**) by the thioacetate group [57], dissolving compound **1** and potassium thioacetate in dry DMF under nitrogen for 5 h at 100 °C to obtain compound **2** in 40% of yield.

Subsequently, the second step consists in the metalation of the obtained compound **2** with Ru_3_(CO)_12_ in toluene under nitrogen for 48 h to obtain compound **3** (yield 75%) [58].

Finally, the third step was the deprotection of the thioacetate, reacting with compound **3** with NaOH in THF for 2 h under nitrogen [59] to yield the corresponding porphyrin thiol derivative and compound **4** in a quantitative yield (>99%).

**Scheme 2:**
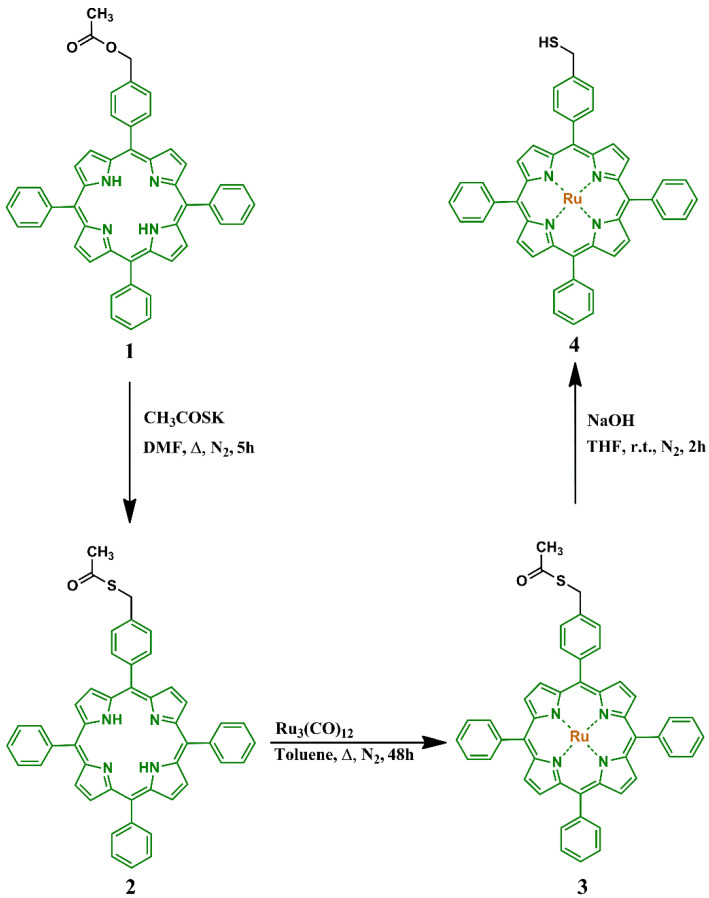
Synthetic pathways for obtaining Ru-TPP-CH_2_-SH (**4**).

In Scheme 2, the synthetic pathways for obtaining the Ru-TPP-CH_2_-SH (**4**) compound are reported.

#### 2.2.3. Synthesis of Ru-TPP-CH_2_S-AuNPs (**5**)

**Scheme 3:**
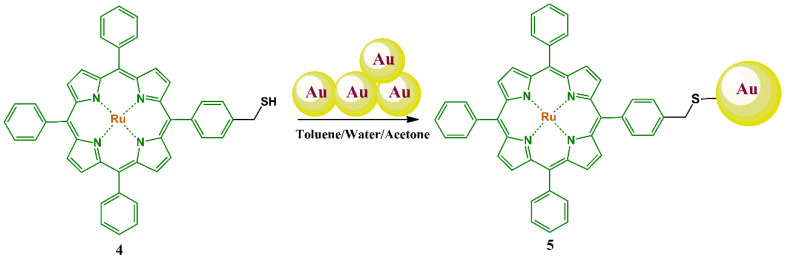
Synthetic pathway for obtaining Ru-TPP-CH_2_S-AuNPs (**5**).

The functionalization of AuNPs with Ru-TPP-CH_2_-SH porphyrin (**4**) was performed by a variant of the two-phases synthetic procedure previously proposed [54,60] and reported in Scheme 3.

Specifically, 2.5 mL of the solution containing AuNPs, stabilized with citrate and 0.6 mL of acetone used as transfer agent, was added to 1 mg/mL of compound **4** dispersed in toluene. The mixture was stirred vigorously until the color changed from red to colorless, indicating that Ru-TPP-CH_2_-SH porphyrins (**4**) were anchored on the surface of the AuNPs to form Ru-TPP-CH_2_S-AuNPs’ final compound (**5**).

### 2.3. Typical Oligomerization Reaction

In order to evaluate the performance of Ru-TPP-CH_2_-SH (**4**) as a catalyst, in a one-neck flask, 1 mg (0.0013 mmol) of Ru-TPP-CH_2_-SH (**4**) was added to 1 mL of substrate (phenylacetylene) under magnetic stirring at 160 °C.

In order to analyze the products of the reaction by gas chromatographic analysis, three aliquots of the mixture were taken up at different reaction times, 3 h, 10 h, 24 h, and 48 h.

The same procedure was repeated by adopting the same reaction condition but using AuNPs and Ru-TPP-CH_2_S-AuNPs (**5**) as catalysts.

The stability of the Ru-TPP-CH_2_S-AuNPs (**5**) system at high reaction temperatures (160 °C) was verified by the UV-Vis method. In fact, after 24 h and 48 h of reaction, no trace of free metalloporphyrin was detected in the reaction medium.

### 2.4. Recycling of the Ru-TPP-CH_2_S-AuNPs (**5**) Catalyst

At the end of the reaction, the solvent was evaporated under vacuum, and the solid was washed with three portions of fresh chloroform or dichloromethane, each time using centrifugation to obtain the starting solid. The absence of any free catalyst in the solution was confirmed by UV-Vis analysis.

The recovered catalyst was dried under vacuum at 60 °C for 2 h and reused.

## 3. Results and Discussion

The synthesized Ru-TPP-CH_2_S-AuNPs (**5**) catalyst was characterized with different techniques. In particular, the average size of AuNPs and the presence of the Ru-TPP-CH_2_-SH porphyrins (**4**) on AuNPs’ surface were estimated by using SEM microscopy. TGA was also used to confirm the obtained functionalization of compound **4** on Au nanoparticles. Subsequently, gas-chromatography analysis was used to confirm the ability of Ru-TPP-CH_2_S-AuNPs (**5**) to be used as a catalyst in oligomerization reactions of phenylacetylenes.

### 3.1. Characterization of Ru-TPP-CH_2_S-AuNPs (**5**) Catalyst

**Figure 1:**
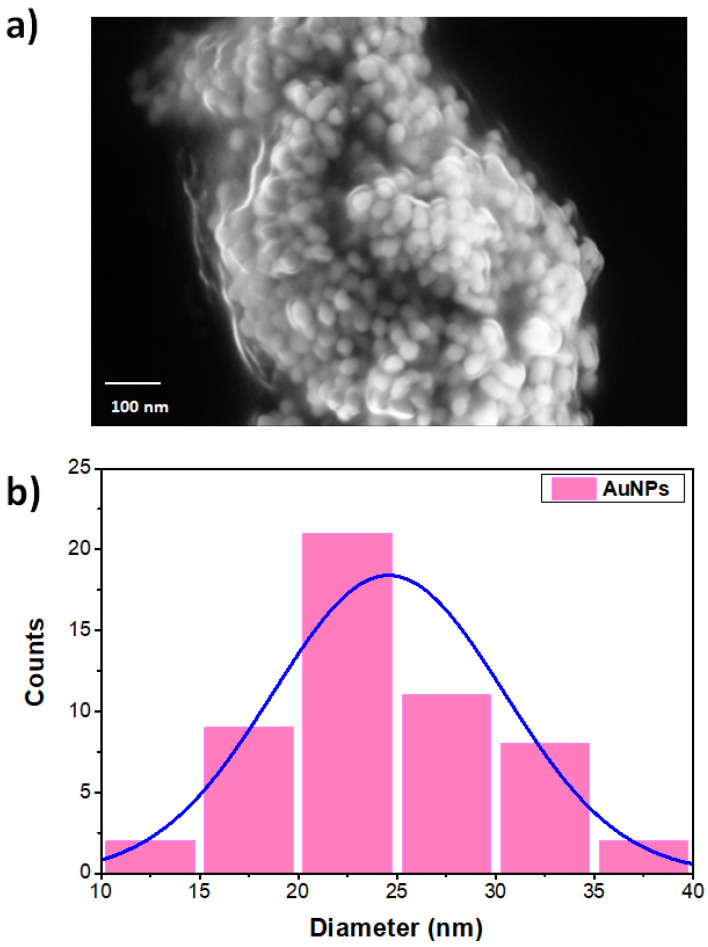
(**a**) SEM micrograph of Ru-TPP-CH_2_S-AuNPs (**5**) and (**b**) particle size distribution of AuNPs.

In Figure 1a, the SEM image of the Ru-TPP-CH_2_S-AuNPs (**5**) catalyst is reported. The size distribution of AuNPs is estimated using SEM analysis showing an average diameter of 24.58 ± 5.85 nm, as reported in Figure 1b. The covalent anchoring of Ru-TPP-CH_2_-SH (**4**) on AuNPs’ surface can be observed in the ~4 nm texture surrounding the AuNPs, as shown in Figure 1a.

**Figure 2:**
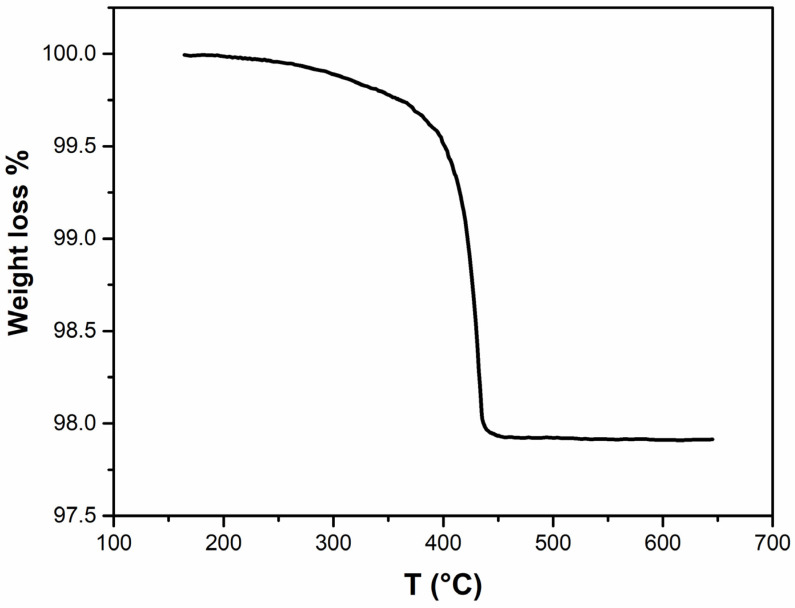
TGA of Ru-TPP-CH_2_S-AuNPs compound (**5**).

The binding of Ru-TPP-CH_2_-SH porphyrins (**4**) to AuNPs’ surface was confirmed by thermogravimetric analysis. In particular, the TGA thermogram of Ru-TPP-CH_2_S-AuNPs, in the range of 100–650 °C, as reported in Figure 2, indicates a weight loss of about 2.1 wt % completed at 430 °C and attributed to the loss of organic moieties, i.e., the porphyrins, bonded to the Au surface. The degree of functionalization of the AuNPs can be estimated by combining SEM and TGA information. More in detail, considering that the density of bulk face-centered cubic (fcc) Au is 59 atoms/nm^3^ and that the average diameter of a gold particle is D ≅ 25 nm, the approximate number of gold atoms in a nanoparticle is consistent with N_Au_ = (59 nm^3^)(π/6) D^3^ = 482,692. Since AuNPs (atomic mass ≅ 200) represent 97.9% of weight loss and ligands (Ru-TPP-CH_2_SH, molar mass 753 g/mol) represent 2.1% of the weight loss, proportionally, this corresponds to an approximate number of 2746 ligands per AuNP.

### 3.2. Oligomerization of Phenylacetylenes Catalyzed by Ru-TPP-CH_2_S-AuNPs

Our investigation was then directed to the evaluation of the efficiency of Ru-TPP-CH_2_S-AuNPs (**5**) as a catalyst for the phenylacetylene oligomerization reaction.

In these regards, the formation of several triphenylbenzenes and 1-phenylnapthalene has been particularly considered.

**Scheme 4:**
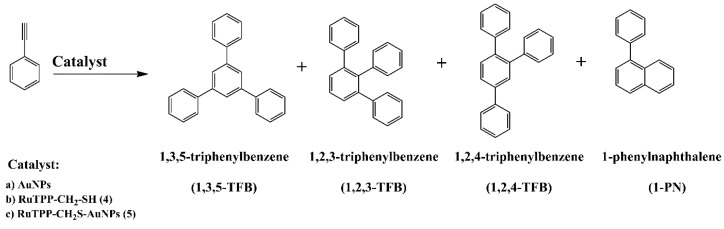
Phenylacetylene oligomerization reaction catalyzed by (a) AuNPs, (b) RuTPP-CH_2_-SH (**4**), and (c) RuTPP-CH_2_S-AuNPs (**5**).

For this instance, a decision was made to study the performance of our new Ru-TPP-CH_2_S-AuNPs (**5**) catalyst in the oligomerization reaction of phenylacetylene using gas chromatographic analysis and to compare the results with those obtained using two other catalysts, i.e., AuNPs and the Ru-TPP-CH_2_-SH (**4**) compound, for the same reaction as shown in Scheme 4.

In Table 1, the formation of dimers and trimers in terms of percentage of product (%) after 24 h and 48 h using AuNPs, Ru-TPP-CH_2_-SH (**4**), and Ru-TPP-CH_2_S-AuNPs (**5**) as catalyst are reported.

Analyzing the results obtained by gas chromatographic analysis during the time interval between 3 h and 10 h for the phenylacetylene oligomerization reaction, this elapsed time is not sufficient to form the expected dimerization/trimerization products. Only low percentages of 1-PN product were obtained for all catalysts used.

The reaction times of 24 h and 48 h are chosen based on previous experimental evidence in which a similar catalyst, i.e., Ru-porphyrin bound to a Merrifield resin [26], was used for the oligomerization reaction. For this reason, the data after 24 h and 48 h, using AuNPs, Ru-TPP-CH_2_-SH (**4**), and Ru-TPP-CH_2_S-AuNPs (**5**) as catalysts, are considered significant and are reported in Table 1.

The control experiment without any catalysts was performed, but no results in terms of the formation of dimers and trimers were obtained.

**Figure 3:**
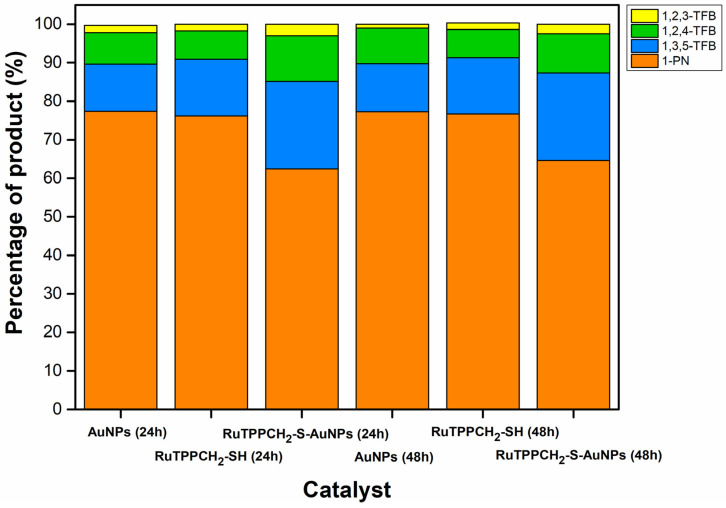
Histogram comparing the GC results of AuNPs, Ru-TPP-CH_2_SH (**4**), and Ru-TPP-CH_2_S-AuNPs (**5**) on the percentage of products (%) of dimers/trimers after 24 h and 48 h.

Figure 3 shows the comparison of percentage of products (%) obtained using AuNPs, Ru-TPP-CH_2_-SH (**4**), and Ru-TPP-CH_2_S-AuNPs (**5**) as catalysts after 24 h and 48 h reaction times.

In particular, by comparing the efficiency of Ru-TPP-CH_2_S-AuNPs (**5**) with respect to AuNPs and Ru-TPP-CH_2_-SH (**4**) at 24 h and 48 h, it can be observed that the functionalization of porphyrin with AuNPs increases, in all cases, the response in terms of percentage of products (%) in the formation of trimers.

Specifically, an evident increase in the percentage of products (%) of 1,3,5-TFB is observed at 24 h: from 12.5% (AuNPs) and 14.7% (Ru-TPP-CH_2_-SH) to 22.7% using Ru-TPP-CH_2_S-AuNPs (**5**) as the catalyst.

A comparable increase, in terms of percentage of product (%) in the formation of trimers, was observed at 48 h using the Ru-TPP-CH_2_S-AuNPs (**5**) system.

Substrate conversion for all reactions reported is always above 95%, and the reaction products did not change under experimental conditions, after three recycling cycles, and remained within the experimental error.

The different formation of dimers and trimers seems to be affected by the presence of AuNPs in terms of an increase in trimer products. This effect can be due to the different steric hindrances of the Ru-TPP-CH_2_S-AuNPs (**5**) compound compared with metalloporphyrin without AuNPs, as reported in our previous studies [26] for an analog system comprising ruthenium porphyrin bound to a Merrifield resin.

**Scheme 5:**
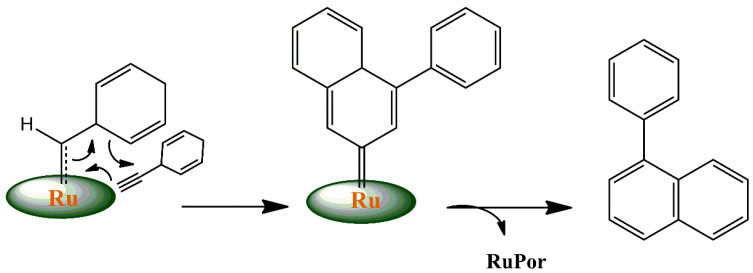
Hypothetical mechanism for the formation of dimerization products.

In a previous study [61], we proposed a mechanism for the oligomerization of phenylacetylene to produce 1-PN, catalyzed by ruthenium porphyrins, in terms of the formation of a vinylidene intermediate of the metal complex by a Z2-1-alkyne-Z1-vinilydene rearrangement. Such an intermediate could then undergo the concerted attack of a second molecule of alkyne in a Diels–Alder reaction (Scheme 5) in order to produce the final dimeric product while the trimers are probably derived from an open intermediate.

## 4. Conclusions

In the present investigation, we synthesized a newly functionalized material by coupling ruthenium porphyrin to AuNPs through the formation of –S–Au bonds using three-step reactions and characterizing the new material by SEM and TGA analysis in order to evaluate the degree of functionalization of AuNPs’ surface. The catalytic efficacy of the new hybrid architecture is studied and compared to the separate moieties in the oligomerization of phenylacetylene. The study determines that compound Ru-TPP-CH_2_S-AuNPs is stable and shows an increase in terms of percentage of product for the formation of trimers for the oligomerization of phenylacetylene, as an effect of the different accessibilities of the catalytic site by the reagent and different electronic properties due to coupling with metal nanoparticles, when compared with metalloporphyrin without AuNPs.

## Figures and Tables

**Table 1:** Oligomerization of phenylacetylene reaction using AuNPs, Ru-TPP-CH_2_-SH (**4**), and Ru-TPP-CH_2_S-AuNPs (**5**) as catalysts.

Reaction Time	AuNPs (%)	Ru-TPP-CH_2_-SH (%)	Ru-TPP-CH_2_S-AuNPs (%)	Products
24 h	77.4	76.1	62.4	1-PN
	1.9	1.7	3.0	1,2,3-TFB
	8.2	7.4	11.9	1,2,4-TFB
	12.5	14.8	22.7	1,3,5-TFB
48 h	77.3	77.0	64.6	1-PN
	1.0	1.7	2.5	1,2,3-TFB
	9.3	7.4	10.2	1,2,4-TFB
	12.4	14.8	22.7	1,3,5-TFB

## Data Availability

The data presented in this study are available on request from the corresponding author on reasonable request.
